# Evaluating the Pharmacological Mechanism of Chinese Medicine Si-Wu-Tang through Multi-Level Data Integration

**DOI:** 10.1371/journal.pone.0072334

**Published:** 2013-11-04

**Authors:** Zhao Fang, Bingxin Lu, Mingyao Liu, Meixia Zhang, Zhenghui Yi, Chengping Wen, Tieliu Shi

**Affiliations:** 1 Center for Bioinformatics and Computational Biology, Shanghai Key Laboratory of Regulatory Biology, the Institute of Biomedical Sciences and School of Life Science, East China Normal University, Shanghai, China; 2 Department of Ophthalmology, West China Hospital, Sichuan University, Chengdu, Sichuan, China; 3 Schizophrenia Program, Shanghai Mental Health Center, Shanghai Jiao Tong University School of Medicine, Shanghai, China; 4 TCM Clinical Basis Institute, Zhejiang University of Chinese Medicine, Hangzhou, Zhejiang, China; 5 Institute of Plant Physiology and Ecology, Shanghai Institutes for Biological Sciences, Chinese Academy of Sciences, Shanghai, China; Semmelweis University, Hungary

## Abstract

Si-Wu-Tang (SWT) is a Traditional Chinese Medicine (TCM) formula widely used for the treatments of gynecological diseases. To explore the pharmacological mechanism of SWT, we incorporated microarray data of SWT with our herbal target database TCMID to analyze the potential activity mechanism of SWT's herbal ingredients and targets. We detected 2,405 differentially expressed genes in the microarray data, 20 of 102 proteins targeted by SWT were encoded by these DEGs and can be targeted by 2 FDA-approved drugs and 39 experimental drugs. The results of pathway enrichment analysis of the 20 predicted targets were consistent with that of 2,405 differentially expressed genes, elaborating the potential pharmacological mechanisms of SWT. Further study from a perspective of protein-protein interaction (PPI) network showed that the predicted targets of SWT function cooperatively to perform their multi-target effects. We also constructed a network to combine herbs, ingredients, targets and drugs together which bridges the gap between SWT and conventional medicine, and used it to infer the potential mechanisms of herbal ingredients. Moreover, based on the hypothesis that the same or similar effects between different TCM formulae may result from targeting the same proteins, we analyzed 27 other TCM formulae which can also treat the gynecological diseases, the subsequent result provides additional insight to understand the potential mechanisms of SWT in treating amenorrhea. Our bioinformatics approach to detect the pharmacology of SWT may shed light on drug discovery for gynecological diseases and could be utilized to investigate other TCM formulae as well.

## Introduction

Traditional Chinese Medicine (TCM) is an ancient system used in disease treatments for several thousand years [1], [2]. Currently, TCM is not only popular in Asia, but also used in United States and Europe as complementary or alternative medicine [3], [4]. Up to now, nearly 100,000 TCM formulae have been recovered [5], [6], each of which normally contains several herbs. Generally, a TCM formula exerts its therapeutic effects through interactions between herbal ingredients and dysfunctional proteins related to the diseases. These ingredients target many molecules in the cell and function cooperatively to increase the therapeutic efficacy and reduce adverse effects of the TCM [6], [7]. Although great efforts have been made to unveil the mechanisms of TCM formulae, the mechanisms of most formulae are still unknown [6], [8].

Because a TCM formula contains many non-effective and needless ingredients, a new approach which combines only active ingredients in one formula has been suggested for new formula discovery [7]. This method is useful for the modernization of TCM because if a formula is simplified to only contain active ingredients, the production of this new formula will rely less on cultivations of herbs and can be manufactured based on methodology of highly-developed chemical synthesis. However, few formulae were simplified by this way as active ingredients of most formulae were still unclear.

Si-Wu-Tang (SWT), composed of four herbs, Radix Rehmanniae Praeparata, Radix Angelicae Sinensis, Rhizoma Ligustici Chuanxiong and Radix Paeoniae Alba [9], is a popular TCM formula widely used for the treatment of gynecological disease in Asia for a long time [10]. It has been reported in the treatment of menstrual discomfort, climacteric syndrome, peri- or postmenopausal syndrome and other estrogen-related diseases [11]–[15]. Besides, this formula also provides the pharmacological effects of anti-inflammatory, vasodilatation and hematopoiesis [16], [17].

Microarray experiment has been conducted to analyze the mechanism of SWT treatment at gene level, suggesting that SWT has a phytoestrogenic effect and act as an Nrf2 activator [6]. However, the results gained from microarray experiment are not convictive enough because the up/down-regulation of mRNA may not lead to a consistent alteration of protein expression [6], [18], [19]. To further investigate the potential mechanism of SWT on disease treatment, we integrated the microarray expression data with the herbal targets obtained from our TCMID database [20]. TCMID is an integrative database that contains data of herbal ingredients, herbal targets, disease-related gene or proteins, drugs and their targets, many of which were collected through text mining. These data can be effectively applied to complement the results of high throughput experiments.

In particular, we want to check whether genes differentially expressed in cells treated with SWT finally lead to therapeutic effects on protein level. Thus we conducted this study by firstly identifying intersections between symbols of previously known targets of the four herbs in SWT in TCMID database and the differentially expressed genes (DEGs), resulting in 20 predicted targets of SWT. Then pathway enrichment analysis and protein-protein interaction network were utilized to explore SWT's pharmacological effects on gynecological diseases. We further identified compounds (herb ingredients and drugs) that may have potential to become new drugs or drugs that may have new therapeutic effects. Subsequently, a novel herb-ingredient-target-drug network was constructed to visually show the relationships between herbs, ingredients, targets and drugs. We also collected other TCM formulae which have therapeutic effects on gynecological diseases and investigated whether these formulae target most of these 20 predicted targets of SWT as well.

## Materials and Methods

### 2.1. Materials

Microarray data of human breast cancer MCF-7 cells treated with SWT were downloaded from Gene Expression Omnibus (GEO: GSE23610), which consist of 54,675 probe sets. Protein targets of each herb constituting SWT (Radix Rehmanniae Praeparata, Radix Angelicae Sinensis, Rhizoma Ligustici Chuanxiong and Radix Paeoniae Alba) identified experimentally from previously published literature as well as TCM formulae with same or similar effects as SWT were obtained from TCMID by searching herbal names and names of all the gynecological diseases respectively [20]. The target names of four herbs in SWT were also retrieved from TCMID. As for other TCM formulae used for treating gynecological diseases, we obtained 27 formulae which mainly treat menstrual discomfort and climacteric syndromes from our TCMID database (Table S1).

### 2.2. Methods

#### 2.2.1. Identification of differentially expressed genes and their intersections with herbal targets

In our study, only gene expression profiles of MCF-7 cells treated with SWT in high concentration was used for the identification of differentially expressed genes, because SWT with high concentration has the similar expression profile to Estradiol treatment on MCF-7 cells and is considered as the effective formula for disease treatment in clinical practices [6]. We compared the SWT group in high concentration with control group and obtained differentially expressed genes by setting p-value <0.05 and fold change >1.5 which is consistent with previous study [6].

To identify the potential targets of each herb in SWT, we used each herb's name to query TCMID, and then retrieved the targets for each ingredient in the herb. In total, we obtained 102 non-redundant targets for all of the identified ingredients of SWT (Table S2). For the 102 targets of SWT in TCMID, we firstly obtained their symbols in HUGO Gene Nomenclature Committee (HGNC) by searching the names of targets [21], and then computed intersections between symbols of these targets and names of the differentially expressed genes to get predicted targets of SWT whose encoding genes are differentially expressed.

#### 2.2.2. Pathway enrichment analysis

We carried out pathway enrichment analysis for these differentially expressed genes using ClueGO (a plugin of Cytoscape) [22] and obtained the pathways enriched with these differentially expressed genes (p<0.05 as the threshold). Pathway enrichment analysis for the predicted protein targets of SWT was also identified in a similar way.

#### 2.2.3. Network construction

Based on the data of protein-protein interactions in HPRD [23] and STRING [24], we constructed a PPI network for predicted targets of SWT with Cytoscape [25] using all the data of protein-protein interactions in HPRD and STRING. Then we identified those interactions directly between the predicted proteins or bridged by only one intermediate protein.

To construct a herb-ingredient-target-drug network, we first selected those ingredients, each of which targets at least one of the predicted targets of SWT. Next we downloaded all the drug names and their target names from DrugBank [26], followed by selecting drugs which also target at least one of the predicted targets of SWT. Finally we constructed the herb-ingredient-target-drug network based on the interactions between ingredients (or drugs) and targets using Cytoscape.

#### 2.2.4. Analysis of other TCM formulae with similar effects as SWT

To analyze the 27 other TCM formulae which can also treat gynecological diseases, we firstly obtained the targets of these formulae in TCMID by searching the names of the herbs in each formula. Subsequently we removed the redundant targets in each formula and retained about 513 unique targets (Table S6) for all 27 formulae. Then we calculated the numbers of occurrence in the 27 TCM formulae for all the 513 targets. To identify the common therapeutic effects among these formulae, we counted the numbers of occurrence for the predicted targets of SWT. To explore the connection between gynecological diseases and the predicted targets, we detected the co-occurrence between each target name and each name of gynecological diseases (such as menstrual discomfort and climacteric syndrome) with google scholar. Through text mining, we found that considerable amount of literatures describe the relationships between the predicted targets and the related gynecological diseases.

## Results

### 3.1. Pathway enrichment analysis for differentially expressed genes

To validate the results from a previous study [6], and explore the potential mechanisms of SWT, we looked for differentially expressed genes in downloaded microarray data, followed by pathway enrichment analysis. In all, we obtained 2,405 differentially expressed genes, corresponding to 3,950 probe sets in the microarray. Pathway enrichment analysis of 2,405 differentially expressed genes showed that these genes were enriched in 7 pathways with p-values less than 0.05 (**Table 1**).

**Table 1:** Seven pathways enriched based on differentially expressed genes with p-values less than 0.05.

No.	Pathway name	P-value
1	Pathways in cancer	1.61E-05
2	Ribosome biogenesis in eukaryotes	8.91E-04
3	p53 signaling pathway	0.006777907
4	Endocytosis	0.011224262
5	Neuroactive ligand-receptor interaction	0.011869812
6	TGF-beta signaling pathway	0.030380161
7	Oxidative Stress Induced Gene Expression Via Nrf2	0.041972061

Consistent with the previous results [6], we found that Nrf2 was significantly impacted by SWT. Furthermore, our research showed that TGF-β signaling pathway was enriched by 24 differentially expressed genes (Table S3). Besides, TGF-β, which plays an important role in this pathway, has been proved to be involved in a variety of physiological processes and many diseases. It was reported that TGF-β had close relationships with osteoporosis and coronary heart disease in postmenopausal women suffering climacteric syndromes [27]–[29]. Since SWT can treat climacteric syndrome and it significantly down-regulates TGF-β coding gene (one of the 2,045 differentially expressed genes, Table S3), we inferred that one of the therapeutic effects of SWT on osteoporosis and coronary heart disease could attribute to the down-regulation of TGF-β by SWT [30]. In other word, pathway enrichment analysis of differentially expressed genes obtained from microarray experiments suggests the potential mechanisms of SWT on treatment of climacteric syndrome.

### 3.2. Pathway enrichment analysis of the20 predicted targets

By focusing on herbal targets of SWT whose encoding genes are differentially expressed, we detected 20 intersections between previously known protein targets of the four herbs of SWT in TCMID and differentially expressed genes, which are 20 predicted targets of SWT (**Table 2**) and used for further study.

**Table 2:** 20 predicted targets of SWT.

No.	Target	Symbol
1	Vascular endothelial growth factor A	VEGFA
2	Telomerase protein component 1	TEP1
3	Mitogen-activated protein kinase 1	MAPK1
4	Interleukin-8	IL8
5	Interleukin-6	IL6
6	Intercellular adhesion molecule 1	ICAM1
7	Proto-oncogene c-Fos	FOS
8	Eukaryotic translation initiation factor 6	EIF6
9	Cytochrome P450 1A1	CYP1A1
10	Cyclin-dependent kinase inhibitor 1	CDKN1A
11	Cyclin-A2	CCNA2
12	Caspase-3	CASP3
13	Transcription factor AP-1	JUN
14	Activator of 90 kDa heat shock protein ATPase homolog 1	AHSA1
15	Serine/threonine-protein kinase Sgk3	SGK3
16	Heparan sulfate glucosamine 3-O-sulfotransferase 3A1	HS3ST3A1
17	Ubiquitin carboxyl-terminal hydrolase isozyme L1	UCHL1
18	Solute carrier family 22 member 5	SLC22A5
19	Choline-phosphate cytidylyltransferase A	PCYT1A
20	Protein CBFA2T1	RUNX1T1

Pathway enrichment analysis of the 20 predicted targets showed that predicted targets of SWT enriched in 40 pathways with p-values less than 0.05. We ranked these pathways according to the p-value of each pathway in an ascending order. The top 20 pathways are shown in **Table 3** **.** It is interesting to note that several pathways can further illustrate the possibly pharmacological mechanisms of SWT.

**Table 3:** The top 20 pathways enriched with 20 predicted targets with p-values less than 0.05.

No.	Pathway name	p-value
1	Pertussis	7.21E-07
2	Rheumatoid arthritis	2.89E-06
3	TSP-1 Induced Apoptosis in Microvascular Endothelial Cell	1.92E-05
4	Salmonella infection	7.20E-05
5	Bladder cancer	1.24E-04
6	Toll-like receptor signaling pathway	1.68E-04
7	Chagas disease (American trypanosomiasis)	1.85E-04
8	Cadmium induces DNA synthesis and proliferation in macrophages	1.97E-04
9	Cells and Molecules involved in local acute inflammatory response	3.03E-04
10	Oxidative Stress Induced Gene Expression Via Nrf2	4.39E-04
11	IL 6 signaling pathway	5.21E-04
12	Colorectal cancer	6.00E-04
13	Fc Epsilon Receptor I Signaling in Mast Cells	0.002144985
14	Repression of Pain Sensation by the Transcriptional Regulator DREAM	0.008579493
15	Malaria	0.010027851
16	Legionellosis	0.013315141
17	NOD-like receptor signaling pathway	0.014802808
18	B Cell Survival Pathway	0.018475697
19	D4-GDI Signaling Pathway	0.018475697
20	Pertussis toxin-insensitive CCR5 Signaling in Macrophage	0.021522095

The pathway of “oxidative stress induced gene expression via Nrf2” (ranked 10), which was also enriched by differentially expressed genes, plays an important role in radio-resistance [31]. It was reported that γ-irradiation-induced formation of protein carbonyls was significantly higher in Nrf2-depleted lung cancer cells, and the increased lethality of ionizing radiation in the absence of Nrf2, suggesting Nrf2 has a constitutive activation to protect against ionizing radiation toxicity and confer radio-resistance [31]. Besides, Nrf2 was suggested to be used as a target of chemopreventive agent. Our pathway enrichment analysis showed that three targets of SWT were enriched in this pathway, including proto-oncogene c-fos (FOS), transcription factor AP-1 (JUN), and mitogen-activated protein kinase 1 (MAPK1). As SWT was reported to have a significant effect on radio-resistance [32]–[35], we inferred that the potential effect of radio-resistance was provided by ingredients in the four herbs of SWT targeting these proteins.

With respect to the pathway of “repression of pain sensation by the transcriptional regulator DREAM” (ranked 14), in general, the opioid receptors modulate pain signaling in response to endogenous peptide ligands and opiate drugs such as morphine [36]. Specifically the kappa opioid receptor plays a key role in the profound analgesia of opiates and is activated by the endogenous peptide ligand dynorphin, encoded by the prodynorphin gene. Production of prodynorphin is transcriptionally regulated by a downstream regulatory element (DRE) in the prodynorphin gene. A transcription factor called DREAM (DRE antagonistic modulator) binds to the DRE and represses prodynorphin transcription [36], [37]. The regulation of prodynorphin expression by DREAM leads to the hypothesis that DREAM is involved in pain signaling. Our research showed that differentially expressed genes which encode two targets of SWT, FOS and JUN, enriched in this pathway. The two protein targets interact with DREAM in this pathway to regulate the expression of preprodynorphin [38] and function as third messengers in the signal transduction mechanisms of pain processes [39]. As SWT was also reported to have a significant therapeutic effect on dysmenorrhea [40]–[43], we proposed that SWT may play its therapeutic role on dysmenorrhea by targeting FOS and JUN to regulate the signaling pathway of pain.

In addition, differentially expressed genes which encode targets of SWT were also enriched in three pathways relating to hematopoiesis, erythropoiesis and leukopoiesis, which are “regulation of hematopoiesis by cytokines” (ranked 25), “EPO signaling pathway” (ranked 32) and “TPO signaling pathway” (ranked 35). This finding provides a meaningful explanation for the significant effect of SWT on treating “blood deficiency”, since SWT can prevent the symptoms of blood deficiency through these pathways, such as decreasing erythrocyte and leukocyte [9], [32], [33].

### 3.3. Protein-protein interaction network analysis

**Figure 1:**
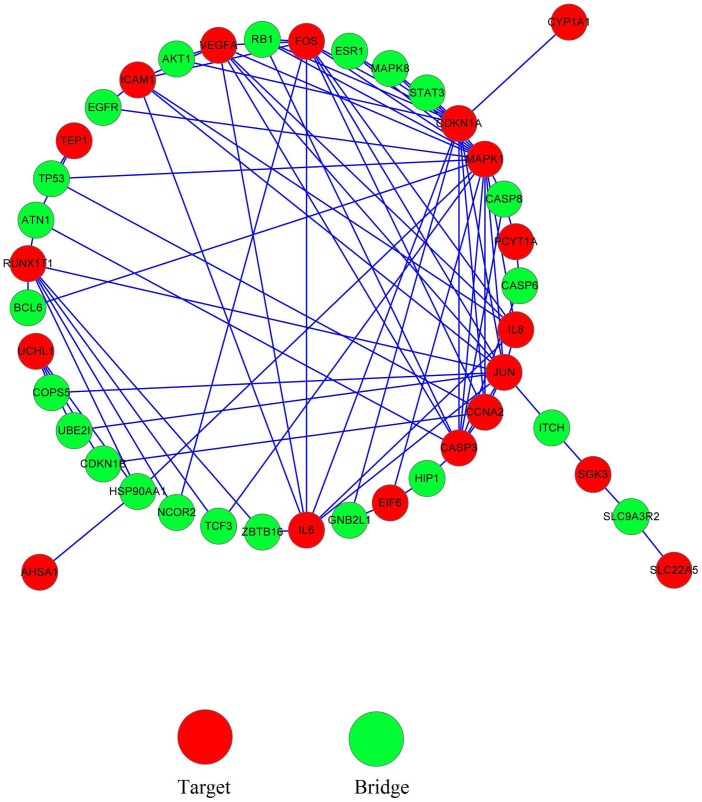
PPI network of 20 predicted herbal targets of SWT.

As shown in **Figure 1**, the 20 predicted protein targets of SWT in the PPI network connected with each other through direct interaction or bridged by an intermediate protein. 19 out of 20 predicted targets were involved in this network. Five proteins, MAPK1, JUN, CDKN1A, CASP3 and FOS were supposed to be hub proteins because each of them connects with at least 10 predicted targets of SWT or intermediate proteins. Particularly, JUN and FOS participated in 28 and 25 of pathways enriched by differentially expressed genes encoding 20 predicted targets of SWT respectively, suggesting that JUN and FOS function as the main targets of SWT and are the potential targets of new drugs.

### 3.4. Herbal ingredients targeting 20 predicted targets of SWT

Since the 102 targets of SWT are targeted by ingredients in the four herbs of SWT, we further selected the ingredients, each of which targets at least one of 20 predicted targets of SWT. For each ingredient with defined targets, it could be a candidate for new drug [44]. The herbs and ingredients which interact with predicted targets of SWT are shown in Table S4.

As shown in Table S4, SWT targets predicted proteins via 16 non-overlapping active ingredients, implying that these 16 ingredients may provide main pharmacological effects of SWT. Accordingly, the four herbs of SWT could potentially be simplified to these 16 ingredients and mass-produced by chemical synthesis, which still need further validation.

### 3.5. Drugs targeting 20 predicted targets of SWT

Generally, if drugs and TCM formulae target the same proteins, they may have same or similar therapeutic effects, which offer an effective way to connect TCM and conventional medicine. Here, to check whether the predicted targets of SWT are targeted by drugs as well, we further searched drugs which target at least one of the 20 predicted targets in DrugBank. As a result, we found 2 FDA-approved small molecule drugs and 39 experimental drugs. These 41 drugs (Table S5) have the potential for treat gynecological diseases, since each of them interacts with at least one of the targets of SWT, and thereby may have some potential for treating similar diseases as SWT does. But this analysis may have low predictive potential because SWT's therapeutic effects are likely the result of its multiple components targeting multiple protein targets, i.e. not a single component targeting a single target.

### 3.6. Herb-ingredient-target-drug network analysis

**Figure 2:**
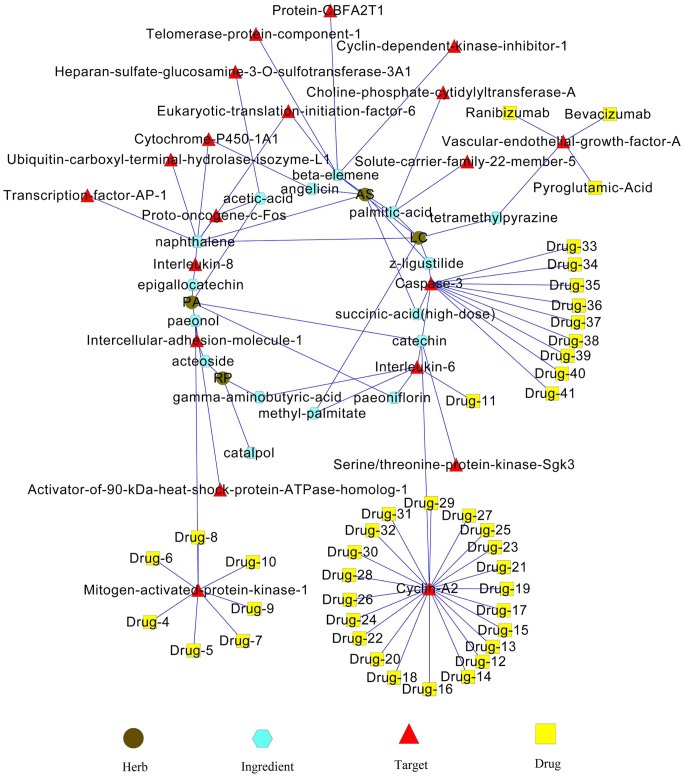
The herb-ingredient-target-drug network for SWT. “PA”, “AS”, “RP”, “LC” represent “Radix Paeoniae Alba”, “Radix Angelicae Sinensis”, “Radix Rehmanniae Praeparata” and “Rhizoma Ligustici Chuanxiong” respectively. “Drug-4”, “Drug-5”, “Drug-6”,..., “Drug-41” represent drugs from “No. 4” to “No. 41” in Table S5 respectively.

Although TCM and conventional medicine are based on different theories, they both provide their therapeutic effects through chemical molecules (herbal ingredients or drugs) targeting proteins (e.g. enzymes) related to the pathological processes of diseases. Thus targets of both herbal ingredients and drugs can bridge the gap between TCM and conventional medicine. Based on this assumption, a network integrating herbs, ingredients, targets and drugs was constructed (**Figure 2**). This network visually shows the relationships between herbs, ingredients, targets and drugs, and is a meaningful attempt in bridging TCM and conventional medicine.

In this network, it is easy to find drugs and ingredients connected by the same targets. For example, vascular endothelial growth factor A (VEGFA), Mitogen-activated protein kinase 1, Cyclin-A2 and Caspase-3 are targeted by many drugs and are acting as hubs to connect SWT and conventional medicine. Specially, VEGFA was simultaneously targeted by three drugs, bevacizumab, ranibizumab and pyroglutamic acid. Besides, VEGFA was also targeted by tetramethylpyrazine, an important ingredient in “Rhizoma Ligustici Chuanxiong”. This connection implies that tetramethylpyrazine could potentially have similar therapeutic effects as these drugs, and therefore has some potential to be considered as a new candidate for therapy. For the other three hubs, the related drugs are still under experimental testing. Thus the potential connections between these experimental drugs and SWT remain to be further verified.

### 3.7. Analysis of TCM formulae with similar effects to SWT

The same or similar therapeutic effects of different TCM formula may be caused by targeting the same targets. Based on this assumption, we checked whether the 27 other formulae treating gynecological diseases retrieved from TCMID also target the 20 predicted targets of SWT (**Materials and Methods**). The numbers of these formulae containing the predicted targets of SWT are summarized in **Table 4**.

**Table 4:** Numbers of occurrence in 27 other TCM formulae for each predicted targets of SWT.

No.	Target	No. of occurrence
1	Caspase-3	27
2	Transcription factor AP-1	27
3	Proto-oncogene c-Fos	27
4	Vascular endothelial growth factor A	25
5	Interleukin-8	25
6	Interleukin-6	25
7	Cyclin-dependent kinase inhibitor 1	25
8	Eukaryotic translation initiation factor 6	23
9	Cytochrome P450 1A1	23
10	Telomerase protein component 1	23
11	Mitogen-activated protein kinase 1	22
12	Intercellular adhesion molecule 1	22
13	Solute carrier family 22 member 5	22
14	Choline-phosphate cytidylyltransferase A	22
15	Protein CBFA2T1	22
16	Ubiquitin carboxyl-terminal hydrolase isozyme L1	21
17	Activator of 90 kDa heat shock protein ATPase homolog 1	19
18	Heparan sulfate glucosamine 3-O-sulfotransferase 3A1	16
19	Cyclin-A2	14
20	Serine/threonine-protein kinase Sgk3	14

As shown in **Table 4**, CAPS3, JUN and FOS were targeted by all the 27 formulae, which probably explain the common mechanism of the 27 formulae having same or similar therapeutic effects as SWT on gynecological diseases. Additionally, VEGFA was targeted by 25 out of 27 TCM formulae. Previous research showed that there was a significant reduction in the expression of VEGFA for women with amenorrhoea [45], whereas our study concerning differentially expressed genes showed that the mRNA of VEGFA was significantly up-regulated under the influence of SWT. Thus it is very likely that SWT can help facilitating the expression of VEGFA. As a result, the symptoms of amenorrhea could be potentially relieved by the increase of VEGFA, which provides the potential pharmacological mechanism for SWT to treat amenorrhea.

## Discussion

TCM formulae typically utilize multi-component therapeutics, similarly drug combination provides increased therapeutic effects by synergisms of two or more drugs and decreased side effects by antagonism, playing a more and more important role in clinical practices and attracting great attention of drug companies and biomedical researchers. Our study also provides a new perspective to find drugs that may provide synergetic effects on treatment of diseases through combinational strategies. For example, if MAPK1 and CASP3, the hub proteins in our constructed PPI network, are targeted by herbal ingredients, the disturbance of the two hub proteins will affect many other proteins in this network and thereby provide same or similar effects as SWT. In addition, as shown in **Figure 2**, MAPK1 and CASP3 can be targeted by 7 and 9 drugs respectively. Therefore, we suppose that drug combinations, such as “Drug-4” and “Drug-33” in **Figure 2** which target MAPK1 and CASP3 respectively, may provide similar therapeutic effects as SWT.

Traditional research methodologies of TCM's pharmacological effects and molecular mechanism are based on the model of single ingredient and single target, which is similar to the rationale applied in modern drug discovery. Thus, methods of natural pharmaceutical chemistry have been widely applied to extract and isolate individual ingredients in herbs. Pharmacological effects of each ingredient are then tested by the methods of modern pharmacology. It is worth noting that individual ingredient may not necessarily have significant therapeutic effects without the coordination with other ingredients, owing to the combinational therapeutic effects for a TCM formula. In other word, the combinational effects of a formula are not necessarily the sum of the individual effect of each ingredient in the formula, which makes it a great challenge for experiments to test the therapeutic effects of TCM [46], [47]. Fortunately, bioinformatic approaches can be applied to this field and to illustrate the potential mechanisms of TCM formulae at the systematic level. In this study, we successfully applied a bioinformatics approach to detect the potential pharmacology of SWT, which may be more reasonable than methods adopted in previous works. For example, the pharmacology of a formula-Qing Luo Yin (QLY) was analysed by Zhang, etc [48]. But they found targets of herbal ingredients based on information of drugs' targets in DrugBank, and this may miss out many targets because only a few herbal ingredients are FDA-approved drugs. In our work, we collected all targets for each herbal ingredients form TCMID and the data was more complete.

According to our results, SWT could be simplified to 16 herbal ingredients. Of course, to verify whether these 16 ingredients can provide similar effects as SWT requires more preclinical experiments. Also, a breast cancer cell line may not be a relevant target cell for observing the effects of drugs on non-cancerous gynecological diseases. Nevertheless, our study demonstrates that the pharmacological effects of TCM formulae can be explored by the integration of multi-level data, such as formulae, herbs, herbal targets, herbal ingredients and drugs as well as PPI network. Our analysis pipeline can also be effectively extended to study mechanisms of other TCM formulae.

## Supporting Information

Table S1 **27 TCM formulae used for treating women's diseases.**
(DOCX)

Table S2 **102 targets of SWT.**
(DOCX)

Table S3 **Symbols of 24 genes enriched in TGF-beta signaling pathway.**
(DOCX)

Table S4 **Herbs and ingredients which target 20 predicted targets of SWT.**
(DOCX)

Table S5 **41 Drugs interacting with 20 predicted targets of SWT.**
(DOCX)

Table S6 **513targets of the 27 formulae.**
(DOCX)
