# Supplementary material for: Evaluating the Pharmacological Mechanism of Chinese Medicine Si-Wu-Tang through Multi-Level Data Integration
Source: PLoS One. 2013 Nov 4;8(11):e72334. doi: 10.1371/journal.pone.0072334 (PMC3817162; doi:10.1371/journal.pone.0072334)
Supplement: Table S1 — 27 TCM formulae used for treating women's diseases. (DOCX) [file pone.0072334.s001.docx]

**Table S1** 27 TCM formulae used for treating women's diseases.

| No. | Formula | Therapeutic effect | URL |
| --- | --- | --- | --- |
| 1 | zhi lian si wu tang | Treating menstrual discomfort | <http://www.megabionet.org/tcmid/prescription/12842/> |
| 2 | ai fu nv zhen wan | Treating irregular  menstrual periods | <http://www.megabionet.org/tcmid/prescription/38893/> |
| 3 | ji kun wan | Treating irregular  menstrual periods | <http://www.megabionet.org/tcmid/prescription/21769/> |
| 4 | nv jin dan | Treating irregular  menstrual periods | <http://www.megabionet.org/tcmid/prescription/44983/> |
| 5 | yu ye jin dan | Treating irregular  menstrual periods | <http://www.megabionet.org/tcmid/prescription/16110/> |
| 6 | an kun zan yu wan | Treating irregular menstruation | <http://www.megabionet.org/tcmid/prescription/27975/> |
| 7 | bao yin jian | Treating irregular menstruation | <http://www.megabionet.org/tcmid/prescription/46497/> |
| 8 | nv jing gao | Treating irregular menstruation | <http://www.megabionet.org/tcmid/prescription/26921/> |
| 9 | wen jing wan | Treating irregular menstruation | <http://www.megabionet.org/tcmid/prescription/30111/> |
| 10 | xiang fu wan | Treating irregular menstruation | <http://www.megabionet.org/tcmid/prescription/8455/> |
| 11 | fu ke jin dan | Treating dysmenorrhea and irregular menstrual periods | <http://www.megabionet.org/tcmid/prescription/17778/> |
| 12 | ba bao kun shun wan | Treating dysmenorrhea and irregular menstruation | <http://www.megabionet.org/tcmid/prescription/23986/> |
| 13 | fu ke shi wei pian | Treating dysmenorrhea and irregular menstruation | <http://www.megabionet.org/tcmid/prescription/45341/> |
| 14 | jing qi fu tong wan | Treating dysmenorrhea and irregular menstruation | <http://www.megabionet.org/tcmid/prescription/18784/> |
| 15 | bai dai wan | Treating dysmenorrheal | <http://www.megabionet.org/tcmid/prescription/17546/> |
| 16 | dao zhi tang | Treating dysmenorrheal | <http://www.megabionet.org/tcmid/prescription/22434/> |
| 17 | fu nv tong jing wan | Treating dysmenorrheal | <http://www.megabionet.org/tcmid/prescription/38832/> |
| 18 | gan cao gan jiang tang | Treating dysmenorrheal | <http://www.megabionet.org/tcmid/prescription/45171/> |
| 19 | hei shen wan | Treating dysmenorrheal | <http://www.megabionet.org/tcmid/prescription/289/> |
| 20 | tao hong si wu tang | Treating dysmenorrheal | <http://www.megabionet.org/tcmid/prescription/44991/> |
| 21 | tong jing zhi tong tang | Treating dysmenorrheal | <http://www.megabionet.org/tcmid/prescription/41746/> |
| 22 | wen jing huo xue tang | Treating dysmenorrheal | <http://www.megabionet.org/tcmid/prescription/641/> |
| 23 | wen jing zhi tong tang | Treating dysmenorrheal | <http://www.megabionet.org/tcmid/prescription/7547/> |
| 24 | xiao ying tang | Treating dysmenorrheal | <http://www.megabionet.org/tcmid/prescription/44926/> |
| 25 | er xian tang | Treating climacteric syndrome | <http://www.megabionet.org/tcmid/prescription/45035/> |
| 26 | qing jin yin xue tang | Treating amenorrhea and advanced menstruation | <http://www.megabionet.org/tcmid/prescription/11779/> |
| 27 | yi mu cao gao | Regulating menstruation | <http://www.megabionet.org/tcmid/prescription/44158/> |
