# Supplementary material for: Evaluating the Pharmacological Mechanism of Chinese Medicine Si-Wu-Tang through Multi-Level Data Integration
Source: PLoS One. 2013 Nov 4;8(11):e72334. doi: 10.1371/journal.pone.0072334 (PMC3817162; doi:10.1371/journal.pone.0072334)
Supplement: Table S2 — 102 targets of SWT. (DOCX) [file pone.0072334.s002.docx]

**Table S2** 102 targets of SWT.

| No. | Targets | UniProt Entry ID |
| --- | --- | --- |
| 1 | Vascular endothelial growth factor A | P15692 |
| 2 | Tyrosinase | P14679 |
| 3 | Cellular tumor antigen p53 | P04637 |
| 4 | Tumor necrosis factor | P01375 |
| 5 | Telomerase protein component 1 | Q99973 |
| 6 | Superoxide dismutase [Mn], mitochondrial | P04179 |
| 7 | Superoxide dismutase [Cu-Zn] | P00441 |
| 8 | Transforming protein RhoA | P61586 |
| 9 | Ras-specific guanine nucleotide-releasing factor 2 | O14827 |
| 10 | Transcription factor p65 | Q04206 |
| 11 | Retinoblastoma-associated protein | P06400 |
| 12 | RAF proto-oncogene serine/threonine-protein kinase | P04049 |
| 13 | Prostaglandin G/H synthase 2 | P35354 |
| 14 | Phosphatidylinositol-3,4,5-trisphosphate 3-phosphatase and dual-specificity protein phosphatase PTEN | P60484 |
| 15 | Peroxisome proliferator-activated receptor gamma | P37231 |
| 16 | Urokinase-type plasminogen activator | P00749 |
| 17 | Protein kinase C beta type | P05771 |
| 18 | Prostaglandin G/H synthase 1 | P23219 |
| 19 | Prostaglandin E2 receptor EP3 subtype | P43115 |
| 20 | NF-kappa-B inhibitor alpha | P25963 |
| 21 | Myeloperoxidase | P05164 |
| 22 | Matrix metalloproteinase-9 | P14780 |
| 23 | Interstitial collagenase | P03956 |
| 24 | Mitogen-activated protein kinase 1 | P28482 |
| 25 | Nitric oxide synthase, inducible | P35228 |
| 26 | Interleukin-8 | P10145 |
| 27 | Interleukin-6 | P05231 |
| 28 | Interleukin-2 | P60568 |
| 29 | Interleukin-1 beta | P01584 |
| 30 | Interleukin-10 | P22301 |
| 31 | Intercellular adhesion molecule 1 | P05362 |
| 32 | Hypoxia-inducible factor 1-alpha | Q16665 |
| 33 | Hyaluronan synthase 2 | Q92819 |
| 34 | Gamma-glutamyltransferase 5 | P36269 |
| 35 | Proto-oncogene c-Fos | P01100 |
| 36 | Fatty acid synthase | P49327 |
| 37 | Neutrophil elastase | P08246 |
| 38 | Eukaryotic translation initiation factor 6 | P56537 |
| 39 | Type I iodothyronine deiodinase | P49895 |
| 40 | Cytochrome P450 3A4 | P08684 |
| 41 | Cytochrome P450 1A1 | P04798 |
| 42 | Cytochrome P450 19A1 | P11511 |
| 43 | Granulocyte-macrophage colony-stimulating factor | P09919 |
| 44 | Choline O-acetyltransferase | P28329 |
| 45 | Cyclin-dependent kinase inhibitor 1 | P38936 |
| 46 | Cell division protein kinase 2 | P24941 |
| 47 | Cell division control protein 2 homolog | P06493 |
| 48 | G2/mitotic-specific cyclin-B1 | P14635 |
| 49 | Cyclin-A2 | P20248 |
| 50 | Catalase | P04040 |
| 51 | Caspase-3 | P42574 |
| 52 | Apoptosis regulator Bcl-2 | P10415 |
| 53 | Apoptosis regulator BAX | Q07812 |
| 54 | Transcription factor AP-1 | P05412 |
| 55 | Amine oxidase [flavin-containing] A | P21397 |
| 56 | Arachidonate 5-lipoxygenase | P09917 |
| 57 | RAC-alpha serine/threonine-protein kinase | P31749 |
| 58 | Activator of 90 kDa heat shock protein ATPase homolog 1 | O95433 |
| 59 | Acetylcholinesterase | P22303 |
| 60 | lipoprotein lipase | P06858 |
| 61 | rennin | N/O |
| 62 | brain-derived neurotrophic factor | P23560 |
| 63 | inositol-3-phosphate synthase 1 | Q9NPH2 |
| 64 | sodium- and chloride-dependent GABA transporter 1 | P30531 |
| 65 | transitional endoplasmic reticulum ATPase | P55072 |
| 66 | poly(ADP-ribose) glycohydrolase | Q86W56 |
| 67 | krueppel-like factor 7 | O75840 |
| 68 | neutrophil cytosol factor 1 | P14598 |
| 69 | serine/threonine-protein kinase Sgk3 | Q96BR1 |
| 70 | pancreatic triacylglycerol lipase | P16233 |
| 71 | catechol O-methyltransferase | P21964 |
| 72 | high affinity immunoglobulin epsilon receptor subunit beta | Q01362 |
| 73 | tissue-type plasminogen activator | P00750 |
| 74 | lipopolysaccharide-binding protein | P18428 |
| 75 | monocyte differentiation antigen CD14 | P08571 |
| 76 | amine oxidase [flavin-containing] B | P27338 |
| 77 | aquaporin-4 | P55087 |
| 78 | heparan sulfate glucosamine 3-O-sulfotransferase 3A1 | Q9Y663 |
| 79 | lysine-specific demethylase NO66 | Q9H6W3 |
| 80 | 6-phosphofructokinase, muscle type | P08237 |
| 81 | non-lysosomal glucosylceramidase | Q9HCG7 |
| 82 | sucrase-isomaltase, intestinal | P14410 |
| 83 | maltase-glucoamylase, intestinal | O43451 |
| 84 | Trehalase | O43280 |
| 85 | lactase-phlorizin hydrolase | P09848 |
| 86 | angiotensin-converting enzyme | Q9BYF1 |
| 87 | p-selectin | Q5R343 |
| 88 | integrin alpha-IIb | P08514 |
| 89 | thromboxane-A synthase | P24557 |
| 90 | beta-nerve growth factor | P01138 |
| 91 | ubiquitin carboxyl-terminal hydrolase isozyme L1 | P09936 |
| 92 | peroxiredoxin-5, mitochondrial | P30044 |
| 93 | rNA-binding protein FUS | P35637 |
| 94 | putative beta-glucuronidase-like protein SMA3 | Q15486 |
| 95 | collagen alpha-1(I) chain | P02452 |
| 96 | Solute carrier family 22 member 5 | O76082 |
| 97 | choline-phosphate cytidylyltransferase A | P49585 |
| 98 | protein CBFA2T1 | Q06455 |
| 99 | neuronal acetylcholine receptor subunit alpha-4 | P43681 |
| 100 | katanin p60 ATPase-containing subunit A1 | O75449 |
| 101 | adenosine receptor A2a | P29274 |
| 102 | Osteopontin | P10451 |
