# Supplementary material for: Evaluating the Pharmacological Mechanism of Chinese Medicine Si-Wu-Tang through Multi-Level Data Integration
Source: PLoS One. 2013 Nov 4;8(11):e72334. doi: 10.1371/journal.pone.0072334 (PMC3817162; doi:10.1371/journal.pone.0072334)
Supplement: Table S3 — Symbols of 24 genes enriched in TGF-beta signaling pathway. (DOCX) [file pone.0072334.s003.docx]

**Table S3** Symbols of 24 genes enriched in TGF-beta signaling pathway.

| No. | Symbol |
| --- | --- |
| 1 | BMP7 |
| 2 | BMPR1A |
| 3 | BMPR1B |
| 4 | BMPR2 |
| 5 | CDKN2B |
| 6 | CUL1 |
| 7 | ID1 |
| 8 | ID2 |
| 9 | ID3 |
| 10 | ID4 |
| 11 | INHBA |
| 12 | LTBP1 |
| 13 | MAPK1 |
| 14 | MYC |
| 15 | PITX2 |
| 16 | PPP2CB |
| 17 | SMAD3 |
| 18 | SMAD6 |
| 19 | SMURF1 |
| 20 | TFDP1 |
| 21 | TGFB2 |
| 22 | TGFBR2 |
| 23 | THBS1 |
| 24 | ZFYVE16 |
