# Supplementary material for: Evaluating the Pharmacological Mechanism of Chinese Medicine Si-Wu-Tang through Multi-Level Data Integration
Source: PLoS One. 2013 Nov 4;8(11):e72334. doi: 10.1371/journal.pone.0072334 (PMC3817162; doi:10.1371/journal.pone.0072334)
Supplement: Table S4 — Herbs and ingredients which target 20 predicted targets of SWT. (DOCX) [file pone.0072334.s004.docx]

**Table S4** Herbs and ingredients which target 20 predicted targets of SWT.

| Herb | Ingredient | Target |
| --- | --- | --- |
| PA | Paeonol | P28482 |
| PA | Epigallocatechin | P10145 |
| PA | Catechin | P05231 |
| PA | Paeoniflorin | P05231 |
| PA | Paeonol | P05362 |
| PA | acetic acid | P01100 |
| PA | Catechin | P20248 |
| PA | Catechin | P42574 |
| PA | Paeonol | O95433 |
| PA | Catechin | Q96BR1 |
| PA | acetic acid | Q9Y663 |
| AS | beta-elemene | Q99973 |
| AS | Naphthalene | P10145 |
| AS | Naphthalene | P01100 |
| AS | Naphthalene | P56537 |
| AS | beta-elemene | P56537 |
| AS | Angelicin | P04798 |
| AS | Naphthalene | P04798 |
| AS | beta-elemene | P38936 |
| AS | z-ligustilide | P42574 |
| AS | succinic acid(high dose) | P42574 |
| AS | Naphthalene | P05412 |
| AS | palmitic acid | O76082 |
| AS | beta-elemene | Q06455 |
| AS | palmitic acid | P49585 |
| AS | Naphthalene | P09936 |
| RP | gamma-aminobutyric acid | P05231 |
| RP | Acteoside | P05362 |
| RP | Catalpol | P42574 |
| LC | Tetramethylpyrazine | P15692 |
| LC | beta-elemene | Q99973 |
| LC | Naphthalene | P10145 |
| LC | methyl palmitate | P05231 |
| LC | Naphthalene | P01100 |
| LC | Naphthalene | P56537 |
| LC | beta-elemene | P56537 |
| LC | Naphthalene | P04798 |
| LC | beta-elemene | P38936 |
| LC | z-ligustilide | P42574 |
| LC | Naphthalene | P05412 |
| LC | palmitic acid | O76082 |
| LC | beta-elemene | Q06455 |
| LC | palmitic acid | P49585 |
| LC | Naphthalene | P09936 |

* "PA", "AS", "RP", "LC" represent "Radix Paeoniae Alba", "Radix Angelicae Sinensis", "Radix Rehmanniae Praeparata" and "Rhizoma Ligustici Chuanxiong" respectively.
