# Supplementary material for: Evaluating the Pharmacological Mechanism of Chinese Medicine Si-Wu-Tang through Multi-Level Data Integration
Source: PLoS One. 2013 Nov 4;8(11):e72334. doi: 10.1371/journal.pone.0072334 (PMC3817162; doi:10.1371/journal.pone.0072334)
Supplement: Table S5 — 41 Drugs interacting with 20 predicted targets of SWT. (DOCX) [file pone.0072334.s005.docx]

**Table S5** 41 Drugs interacting with 20 predicted targets of SWT.

| No. | Drug | Target |
| --- | --- | --- |
| 1 | Bevacizumab | P15692 |
| 2 | Ranibizumab | P15692 |
| 3 | Pyroglutamic Acid | P15692 |
| 4 | N,n-dimethyl-4-(4-phenyl-1h-pyrazol-3-yl)-1h-pyrrole-2-carboxamide | P28482 |
| 5 | N-benzyl-4-[4-(3-chlorophenyl)-1h-pyrazol-3-yl]-1h-pyrrole-2-carboxamide | P28482 |
| 6 | (S)-n-(1-(3-chloro-4-fluorophenyl)-2-hydroxyethyl)-4-(4-(3-chlorophenyl)-1h-pyrazol-3-yl)-1h-pyrrole-2-carboxamide | P28482 |
| 7 | (3r,5z,8s,9s,11e)-8,9,16-trihydroxy-14-methoxy-3-methyl-3,4,9,10-tetrahydro-1h-2-benzoxacyclotetradecine-1,7(8h)-dione | P28482 |
| 8 | 5-(2-phenylpyrazolo[1,5-a]pyridin-3-yl)-1h-pyrazolo[3,4-c]pyridazin-3-amine | P28482 |
| 9 | (1ar,8s,13s,14s,15ar)-5,13,14-trihydroxy-3-methoxy-8-methyl-8,9,13,14,15,15a-hexahydro-6h-oxireno[k][2]benzoxacyclotetradecine-6,12(1ah)-dione | P28482 |
| 10 | [4-({5-(aminocarbonyl)-4-[(3-methylphenyl)amino]pyrimidin-2-yl}amino)phenyl]acetic acid | P28482 |
| 11 | Ginseng | P05231 |
| 12 | 4-[(7-oxo-7h-thiazolo[5,4-e]indol-8-ylmethyl)-amino]-n-pyridin-2-yl-benzenesulfonamide | P20248 |
| 13 | N-(3-cyclopropyl-1h-pyrazol-5-yl)-2-(2-naphthyl)acetamide | P20248 |
| 14 | 2-anilino-6-cyclohexylmethoxypurine | P20248 |
| 15 | O6-cyclohexylmethoxy-2-(4'-sulphamoylanilino) purine | P20248 |
| 16 | (2s)-n-[(3z)-5-cyclopropyl-3h-pyrazol-3-ylidene]-2-[4-(2-oxoimidazolidin-1-yl)phenyl]propanamide | P20248 |
| 17 | N-cyclopropyl-4-pyrazolo[1,5-b]pyridazin-3-ylpyrimidin-2-amine | P20248 |
| 18 | 6-cyclohexylmethoxy-2-(3'-chloroanilino) purine | P20248 |
| 19 | 5-[5,6-bis(methyloxy)-1h-benzimidazol-1-yl]-3-{[1-(2-chlorophenyl)ethyl]oxy}-2-thiophenecarboxamide | P20248 |
| 20 | 4-{5-[(z)-(2-imino-4-oxo-1,3-thiazolidin-5-ylidene)methyl]-2-furyl}-n-methylbenzenesulfonamide | P20248 |
| 21 | 4-{5-[(z)-(2-imino-4-oxo-1,3-thiazolidin-5-ylidene)methyl]furan-2-yl}benzenesulfonamide | P20248 |
| 22 | 4-{5-[(z)-(2-imino-4-oxo-1,3-thiazolidin-5-ylidene)methyl]furan-2-yl}-2-(trifluoromethyl)benzenesulfonamide | P20248 |
| 23 | 4-{5-[(z)-(2-imino-4-oxo-1,3-thiazolidin-5-ylidene)methyl]furan-2-yl}benzoic acid | P20248 |
| 24 | 1-(3,5-dichlorophenyl)-5-methyl-1h-1,2,4-triazole-3-carboxylic acid | P20248 |
| 25 | Hydroxy(oxo)(3-{[(2z)-4-[3-(1h-1,2,4-triazol-1-ylmethyl)phenyl]pyrimidin-2(5h)-ylidene]amino}phenyl)ammonium | P20248 |
| 26 | 4-methyl-5-{(2e)-2-[(4-morpholin-4-ylphenyl)imino]-2,5-dihydropyrimidin-4-yl}-1,3-thiazol-2-amine | P20248 |
| 27 | 6-cyclohexylmethyloxy-2-(4'-hydroxyanilino)purine | P20248 |
| 28 | 4-(6-cyclohexylmethoxy-9h-purin-2-ylamino)—benzamide | P20248 |
| 29 | 3-(6-cyclohexylmethoxy-9h-purin-2-ylamino)-benzenesulfonamide | P20248 |
| 30 | 1-methyl-8-(phenylamino)-4,5-dihydro-1h-pyrazolo[4,3-h]quinazoline-3-carboxylic acid | P20248 |
| 31 | 1-[4-(aminosulfonyl)phenyl]-1,6-dihydropyrazolo[3,4-e]indazole-3-carboxamide | P20248 |
| 32 | 4-{[4-amino-6-(cyclohexylmethoxy)-5-nitrosopyrimidin-2-yl]amino}benzamide | P20248 |
| 33 | 5-[4-(1-carboxymethyl-2-oxo-propylcarbamoyl)-benzylsulfamoyl]-2-hydroxy-benzoic acid | P42574 |
| 34 | 2-hydroxy-5-(2-mercapto-ethylsulfamoyl)-benzoic acid | P42574 |
| 35 | Methyl (3s)-3-[(tert-butoxycarbonyl)amino]-4-oxopentanoate | P42574 |
| 36 | 1-methyl-5-(2-phenoxymethyl-pyrrolidine-1-sulfonyl)-1h-indole-2,3-dione | P42574 |
| 37 | [N-(3-dibenzylcarbamoyl-oxiranecarbonyl)-hydrazino]-acetic acid | P42574 |
| 38 | 4-[5-(2-carboxy-1-formyl-ethylcarbamoyl)-pyridin-3-yl]-benzoic acid | P42574 |
| 39 | (1S)-2-oxo-1-phenyl-2-[(1,3,4-trioxo-1,2,3,4-tetrahydroisoquinolin-5-yl)amino]ethyl acetate | P42574 |
| 40 | (1S)-1-(3-chlorophenyl)-2-oxo-2-[(1,3,4-trioxo-1,2,3,4-tetrahydroisoquinolin-5-yl)amino]ethyl acetate | P42574 |
| 41 | N-[3-(2-fluoroethoxy)phenyl]-N'-(1,3,4-trioxo-1,2,3,4-tetrahydroisoquinolin-6-yl)butanediamide | P42574 |
