# Supplementary material for: Evaluating the Pharmacological Mechanism of Chinese Medicine Si-Wu-Tang through Multi-Level Data Integration
Source: PLoS One. 2013 Nov 4;8(11):e72334. doi: 10.1371/journal.pone.0072334 (PMC3817162; doi:10.1371/journal.pone.0072334)
Supplement: Table S6 — 513targets of the 27 formulae. (DOCX) [file pone.0072334.s006.docx]

**Table S6** 513targets of the 27 formulae.

| No. | Target |
| --- | --- |
| 1 | Nuclear receptor subfamily 1 group I member 2 |
| 2 | Cytochrome P450 3A4 |
| 3 | Cellular tumor antigen p53 |
| 4 | Cyclin-dependent kinase inhibitor 1 |
| 5 | Fatty acid synthase |
| 6 | Matrix metalloproteinase-9 |
| 7 | NF-kappa-B inhibitor alpha |
| 8 | Transcription factor p65 |
| 9 | Transcription factor AP-1 |
| 10 | Proto-oncogene c-Fos |
| 11 | Nucleophosmin |
| 12 | Endothelin-converting enzyme 1 |
| 13 | Endothelin-1 |
| 14 | Endothelin-1 receptor |
| 15 | Poly [ADP-ribose] polymerase 4 |
| 16 | Cytochrome P450 1A1 |
| 17 | Activator of 90 kDa heat shock protein ATPase homolog 1 |
| 18 | Apoptosis regulator Bcl-2 |
| 19 | Myc proto-oncogene protein |
| 20 | Calcitonin receptor |
| 21 | Proto-oncogene tyrosine-protein kinase Src |
| 22 | Integrin beta-3 |
| 23 | Cytochrome P450 1A2 |
| 24 | Caspase-3 |
| 25 | Collagen alpha-1(I) chain |
| 26 | Collagen alpha-1(III) chain |
| 27 | Transforming growth factor beta-1 |
| 28 | Metalloproteinase inhibitor 1 |
| 29 | Superoxide dismutase [Cu-Zn] |
| 30 | Atrial natriuretic factor |
| 31 | Actin, cytoplasmic 1 |
| 32 | Neurofibromin |
| 33 | Alpha-1A adrenergic receptor |
| 34 | Nitric oxide synthase, endothelial |
| 35 | Ryanodine receptor 2 |
| 36 | Mitogen-activated protein kinase 1 |
| 37 | Protein kinase C beta type |
| 38 | Tissue-type plasminogen activator |
| 39 | Thrombomodulin |
| 40 | Actin, aortic smooth muscle |
| 41 | Spermatogenic leucine zipper protein 1 |
| 42 | 72 kDa type IV collagenase |
| 43 | G1/S-specific cyclin-D1 |
| 44 | Probable E3 ubiquitin-protein ligase HERC5 |
| 45 | RAC-alpha serine/threonine-protein kinase |
| 46 | Eukaryotic translation initiation factor 6 |
| 47 | Prostaglandin G/H synthase 2 |
| 48 | Dual oxidase 2 |
| 49 | Plasminogen activator inhibitor 1 |
| 50 | Vascular endothelial growth factor A |
| 51 | Vascular endothelial growth factor receptor 2 |
| 52 | PRKC apoptosis WT1 regulator protein |
| 53 | Vascular cell adhesion protein 1 |
| 54 | Intercellular adhesion molecule 1 |
| 55 | Collagen alpha-1(VII) chain |
| 56 | G1/S-specific cyclin-D3 |
| 57 | Cell division protein kinase 4 |
| 58 | Cell division protein kinase 2 |
| 59 | Bcl-2-like protein 1 |
| 60 | Caspase-9 |
| 61 | Caspase-7 |
| 62 | T-cell surface glycoprotein CD1a |
| 63 | CD83 antigen |
| 64 | Amyloid beta A4 protein |
| 65 | Signal transducer and activator of transcription 3 |
| 66 | Baculoviral IAP repeat-containing protein 5 |
| 67 | Tumor necrosis factor |
| 68 | Low affinity immunoglobulin epsilon Fc receptor |
| 69 | 3-hydroxy-3-methylglutaryl-coenzyme A reductase |
| 70 | Integrin beta-2 |
| 71 | NADPH--cytochrome P450 reductase |
| 72 | Insulin |
| 73 | Arachidonate 5-lipoxygenase |
| 74 | Interleukin-6 |
| 75 | C5a anaphylatoxin chemotactic receptor |
| 76 | Thromboxane A2 receptor |
| 77 | Catalase |
| 78 | Nitric oxide synthase, inducible |
| 79 | Type I iodothyronine deiodinase |
| 80 | Glutathione S-transferase P |
| 81 | Interleukin-1 beta |
| 82 | Interleukin-8 |
| 83 | Hyaluronan synthase 2 |
| 84 | Poly(ADP-ribose) glycohydrolase |
| 85 | Lipoprotein lipase |
| 86 | Cytochrome P450 19A1 |
| 87 | Krueppel-like factor 7 |
| 88 | Peroxisome proliferator-activated receptor gamma |
| 89 | Neutrophil cytosol factor 1 |
| 90 | Serine/threonine-protein kinase Sgk3 |
| 91 | Pancreatic triacylglycerol lipase |
| 92 | Catechol O-methyltransferase |
| 93 | Granulocyte-macrophage colony-stimulating factor |
| 94 | High affinity immunoglobulin epsilon receptor subunit beta |
| 95 | Ras-specific guanine nucleotide-releasing factor 2 |
| 96 | G2/mitotic-specific cyclin-B1 |
| 97 | Cyclin-A2 |
| 98 | Superoxide dismutase [Mn], mitochondrial |
| 99 | Urokinase-type plasminogen activator |
| 100 | Interstitial collagenase |
| 101 | Lipopolysaccharide-binding protein |
| 102 | Monocyte differentiation antigen CD14 |
| 103 | Interleukin-2 |
| 104 | Amine oxidase [flavin-containing] A |
| 105 | Amine oxidase [flavin-containing] B |
| 106 | Apoptosis regulator BAX |
| 107 | Tyrosinase |
| 108 | Phosphatidylinositol-3,4,5-trisphosphate 3-phosphatase and dual-specificity protein phosphatase PTEN |
| 109 | Aquaporin-4 |
| 110 | Interleukin-10 |
| 111 | Myeloperoxidase |
| 112 | Heparan sulfate glucosamine 3-O-sulfotransferase 3A1 |
| 113 | Lysine-specific demethylase NO66 |
| 114 | Prostaglandin E2 receptor EP3 subtype |
| 115 | Renin |
| 116 | 6-phosphofructokinase, muscle type |
| 117 | Non-lysosomal glucosylceramidase |
| 118 | Sucrase-isomaltase, intestinal |
| 119 | Maltase-glucoamylase, intestinal |
| 120 | Trehalase |
| 121 | Lactase-phlorizin hydrolase |
| 122 | Angiotensin-converting enzyme |
| 123 | P-selectin |
| 124 | Gamma-glutamyltransferase 5 |
| 125 | Inositol-3-phosphate synthase 1 |
| 126 | Retinoblastoma-associated protein |
| 127 | Cyclin-dependent kinase inhibitor 2A, isoforms 1/2/3 |
| 128 | NTF2-related export protein 1 |
| 129 | Transcription factor Sp1 |
| 130 | Ectonucleotide pyrophosphatase/phosphodiesterase family member 7 |
| 131 | Ubiquitin carboxyl-terminal hydrolase isozyme L1 |
| 132 | Peroxiredoxin-5, mitochondrial |
| 133 | RNA-binding protein FUS |
| 134 | RAF proto-oncogene serine/threonine-protein kinase |
| 135 | Scavenger receptor class B member 1 |
| 136 | Glucagon |
| 137 | Tripartite motif-containing protein 26 |
| 138 | Proliferating cell nuclear antigen |
| 139 | Hepatocyte nuclear factor 1-alpha |
| 140 | Hepatocyte nuclear factor 4-alpha |
| 141 | Serum albumin |
| 142 | BMP-binding endothelial regulator protein |
| 143 | Mitochondrial uncoupling protein 2 |
| 144 | Calmodulin |
| 145 | Tumor necrosis factor receptor superfamily member 5 |
| 146 | T-lymphocyte activation antigen CD80 |
| 147 | T-lymphocyte activation antigen CD86 |
| 148 | Putative beta-glucuronidase-like protein SMA3 |
| 149 | Solute carrier family 22 member 5 |
| 150 | Choline-phosphate cytidylyltransferase A |
| 151 | Protein CBFA2T1 |
| 152 | Transforming protein RhoA |
| 153 | Telomerase protein component 1 |
| 154 | Cell division control protein 2 homolog |
| 155 | Katanin p60 ATPase-containing subunit A1 |
| 156 | Adenosine receptor A2a |
| 157 | Acetylcholinesterase |
| 158 | Choline O-acetyltransferase |
| 159 | Osteopontin |
| 160 | Prostaglandin G/H synthase 1 |
| 161 | Integrin alpha-IIb |
| 162 | Hypoxia-inducible factor 1-alpha |
| 163 | Thromboxane-A synthase |
| 164 | Brain-derived neurotrophic factor |
| 165 | Beta-nerve growth factor |
| 166 | Neuronal acetylcholine receptor subunit alpha-4 |
| 167 | Neutrophil elastase |
| 168 | Fos-related antigen 2 |
| 169 | Gamma-aminobutyric acid type B receptor subunit 1 |
| 170 | Junctional adhesion molecule A |
| 171 | E-selectin |
| 172 | Tyrosine-protein kinase JAK2 |
| 173 | Metallothionein-2 |
| 174 | Solute carrier family 2, facilitated glucose transporter member 1 |
| 175 | Estrogen receptor beta |
| 176 | Estrogen receptor |
| 177 | ATP synthase subunit beta, mitochondrial |
| 178 | NADH-ubiquinone oxidoreductase chain 6 |
| 179 | NAD-dependent deacetylase sirtuin-1 |
| 180 | Interleukin-4 |
| 181 | 3 beta-hydroxysteroid dehydrogenase/Delta 5-->4-isomerase type 2 |
| 182 | 3 beta-hydroxysteroid dehydrogenase/Delta 5-->4-isomerase type 1 |
| 183 | Tyrosine-protein kinase BTK |
| 184 | Cystic fibrosis transmembrane conductance regulator |
| 185 | Tyrosine-protein kinase SYK |
| 186 | Procollagen C-endopeptidase enhancer 1 |
| 187 | Signal transducer and activator of transcription 1-alpha/beta |
| 188 | Estrogen sulfotransferase |
| 189 | UDP-glucuronosyltransferase 1-1 |
| 190 | Serine-protein kinase ATM |
| 191 | Serine/threonine-protein kinase Chk2 |
| 192 | Peroxisome proliferator-activated receptor alpha |
| 193 | Low-density lipoprotein receptor |
| 194 | C-C motif chemokine 2 |
| 195 | 15-hydroxyprostaglandin dehydrogenase [NAD+] |
| 196 | Lymphokine-activated killer T-cell-originated protein kinase |
| 197 | Mitotic checkpoint serine/threonine-protein kinase BUB1 |
| 198 | Cell division cycle protein 20 homolog |
| 199 | Aldose reductase |
| 200 | Alpha-crystallin B chain |
| 201 | Transforming growth factor beta-2 |
| 202 | Fibronectin |
| 203 | Androgen receptor |
| 204 | Prostate-specific antigen |
| 205 | M-phase inducer phosphatase 3 |
| 206 | Hsp90 co-chaperone Cdc37 |
| 207 | Histone deacetylase 6 |
| 208 | Dual specificity mitogen-activated protein kinase kinase 5 |
| 209 | Mitogen-activated protein kinase 12 |
| 210 | Apolipoprotein A-I |
| 211 | Serine/threonine-protein kinase PLK1 |
| 212 | E3 ubiquitin-protein ligase CCNB1IP1 |
| 213 | Mediator of DNA damage checkpoint protein 1 |
| 214 | Serine/threonine-protein kinase PLK2 |
| 215 | Scaffold attachment factor B1 |
| 216 | Receptor tyrosine-protein kinase erbB-2 |
| 217 | Probable transcription factor PML |
| 218 | Nuclear receptor corepressor 1 |
| 219 | Glucokinase |
| 220 | ATP-sensitive inward rectifier potassium channel 11 |
| 221 | 1,25-dihydroxyvitamin D(3) 24-hydroxylase, mitochondrial |
| 222 | Prostaglandin E2 receptor EP2 subtype |
| 223 | Prostaglandin F2-alpha receptor |
| 224 | Beta-galactosidase |
| 225 | Glial fibrillary acidic protein |
| 226 | Mesothelin |
| 227 | Epidermal growth factor receptor |
| 228 | E3 ubiquitin-protein ligase Mdm2 |
| 229 | Insulin-like growth factor 1 receptor |
| 230 | Mitogen-activated protein kinase 3 |
| 231 | Insulin-like growth factor IA |
| 232 | Aryl hydrocarbon receptor |
| 233 | Peroxisome proliferator-activated receptor gamma coactivator 1-beta |
| 234 | Somatotropin |
| 235 | Transient receptor potential cation channel subfamily M member 2 |
| 236 | Trefoil factor 1 |
| 237 | Carnitine O-palmitoyltransferase 1, liver isoform |
| 238 | Beta-1,4-galactosyltransferase 4 |
| 239 | Growth hormone receptor |
| 240 | Caveolin-1 |
| 241 | Transforming growth factor beta-1-induced transcript 1 protein |
| 242 | CD5 antigen-like |
| 243 | Protein disulfide-isomerase |
| 244 | Apolipoprotein B-100 |
| 245 | Microsomal triglyceride transfer protein large subunit |
| 246 | Leucyl-tRNA synthetase, cytoplasmic |
| 247 | Steroid 21-hydroxylase |
| 248 | Growth arrest and DNA damage-inducible protein GADD45 alpha |
| 249 | BRCA1-associated RING domain protein 1 |
| 250 | RNA-binding protein 45 |
| 251 | Ubiquitin carboxyl-terminal hydrolase BAP1 |
| 252 | Histone acetyltransferase p300 |
| 253 | DNA repair protein RAD51 homolog 1 |
| 254 | Antigen KI-67 |
| 255 | T-lymphoma invasion and metastasis-inducing protein 2 |
| 256 | Bcl2 antagonist of cell death |
| 257 | Interferon gamma |
| 258 | Mitogen-activated protein kinase 8 |
| 259 | Ephrin type-B receptor 2 |
| 260 | Scavenger receptor cysteine-rich type 1 protein M130 |
| 261 | Cyclic AMP-responsive element-binding protein 1 |
| 262 | Cytosolic phospholipase A2 |
| 263 | 78 kDa glucose-regulated protein |
| 264 | Heme oxygenase 1 |
| 265 | Mitogen-activated protein kinase 13 |
| 266 | Vascular endothelial growth factor C |
| 267 | Vascular endothelial growth factor receptor 3 |
| 268 | Eukaryotic translation initiation factor 4E |
| 269 | Heparin-binding growth factor 2 |
| 270 | Eukaryotic translation initiation factor 4 gamma 1 |
| 271 | Olfactory receptor 1D2 |
| 272 | Cytochrome P450 2B6 |
| 273 | Trans-acting T-cell-specific transcription factor GATA-3 |
| 274 | Caspase-8 |
| 275 | NF-kappa-B essential modulator |
| 276 | High mobility group protein B1 |
| 277 | Eotaxin |
| 278 | Cytochrome P450 11B2, mitochondrial |
| 279 | Corticosteroid 11-beta-dehydrogenase isozyme 2 |
| 280 | Alanine aminotransferase 2 |
| 281 | Gap junction alpha-1 protein |
| 282 | Catenin alpha-1 |
| 283 | Catenin beta-1 |
| 284 | Junction plakoglobin |
| 285 | Cytochrome P450 2E1 |
| 286 | Glucocorticoid receptor |
| 287 | Bcl-2 homologous antagonist/killer |
| 288 | Bcl-2-related ovarian killer protein |
| 289 | Bcl-2-interacting killer |
| 290 | Bcl-2-modifying factor |
| 291 | BH3-interacting domain death agonist |
| 292 | Telomerase reverse transcriptase |
| 293 | 7-dehydrocholesterol reductase |
| 294 | Putative adenosylhomocysteinase 2 |
| 295 | Phospholipase B1, membrane-associated |
| 296 | Indolethylamine N-methyltransferase |
| 297 | Hormone-sensitive lipase |
| 298 | Insulin-like growth factor-binding protein 1 |
| 299 | Aquaporin-1 |
| 300 | Nuclear factor of activated T-cells, cytoplasmic 3 |
| 301 | Glutathione reductase, mitochondrial |
| 302 | Progonadoliberin-1 |
| 303 | Interleukin-1 alpha |
| 304 | Tumor necrosis factor ligand superfamily member 6 |
| 305 | DNA damage-inducible transcript 3 protein |
| 306 | Alpha-amylase 1 |
| 307 | Chitinase-3-like protein 1 |
| 308 | Nuclear receptor subfamily 1 group I member 3 |
| 309 | Delta-aminolevulinic acid dehydratase |
| 310 | Sterol regulatory element-binding protein 1 |
| 311 | Sterol regulatory element-binding protein 2 |
| 312 | Hexokinase-1 |
| 313 | Pancreatic alpha-amylase |
| 314 | Glutamine synthetase |
| 315 | Glucose-6-phosphatase |
| 316 | Long-chain-fatty-acid--CoA ligase 1 |
| 317 | Long-chain-fatty-acid--CoA ligase 4 |
| 318 | Tyrosine 3-monooxygenase |
| 319 | Aromatic-L-amino-acid decarboxylase |
| 320 | Xanthine dehydrogenase/oxidase |
| 321 | Adenylate cyclase type 2 |
| 322 | DNA topoisomerase 2-alpha |
| 323 | Baculoviral IAP repeat-containing protein 4 |
| 324 | Prostaglandin E synthase |
| 325 | Kinetochore protein Nuf2 |
| 326 | Induced myeloid leukemia cell differentiation protein Mcl-1 |
| 327 | Hepatocyte growth factor receptor |
| 328 | CD40 ligand |
| 329 | DNA topoisomerase 1 |
| 330 | Insulin receptor |
| 331 | Solute carrier family 2, facilitated glucose transporter member 4 |
| 332 | Inositol oxygenase |
| 333 | Glycine receptor subunit alpha-1 |
| 334 | Phosphatidylinositol-5-phosphate 4-kinase type-2 alpha |
| 335 | Platelet glycoprotein 4 |
| 336 | Basic leucine zipper transcriptional factor ATF-like 3 |
| 337 | Multidrug resistance protein 1 |
| 338 | Inhibitor of nuclear factor kappa-B kinase subunit beta |
| 339 | Sodium- and chloride-dependent GABA transporter 1 |
| 340 | Transitional endoplasmic reticulum ATPase |
| 341 | Succinate dehydrogenase [ubiquinone] iron-sulfur subunit, mitochondrial |
| 342 | Stromal cell-derived factor 1 |
| 343 | ATP-binding cassette sub-family G member 2 |
| 344 | Galanin |
| 345 | Cell division control protein 42 homolog |
| 346 | Ras-related C3 botulinum toxin substrate 1 |
| 347 | Proprotein convertase subtilisin/kexin type 9 |
| 348 | Delta-1-pyrroline-5-carboxylate synthase |
| 349 | Angiotensinogen |
| 350 | Early growth response protein 1 |
| 351 | Platelet-derived growth factor subunit A |
| 352 | Wee1-like protein kinase |
| 353 | Cytochrome c |
| 354 | Early activation antigen CD69 |
| 355 | Interleukin-2 receptor subunit alpha |
| 356 | Neuromodulin |
| 357 | Glutathione S-transferase Mu 1 |
| 358 | Glutathione S-transferase Mu 2 |
| 359 | DNA nucleotidylexotransferase |
| 360 | Canalicular multispecific organic anion transporter 1 |
| 361 | Sphingomyelin phosphodiesterase 2 |
| 362 | Natural resistance-associated macrophage protein 2 |
| 363 | Ferritin, mitochondrial |
| 364 | Beta-glucuronidase |
| 365 | Galectin-1 |
| 366 | Medium-chain specific acyl-CoA dehydrogenase, mitochondrial |
| 367 | Cytochrome P450 2B10 |
| 368 | Bone morphogenetic protein 2 |
| 369 | Mothers against decapentaplegic homolog 1 |
| 370 | Transient receptor potential cation channel subfamily M member 8 |
| 371 | Bcl-2-related protein A1 |
| 372 | TNF receptor-associated factor 1 |
| 373 | CASP8 and FADD-like apoptosis regulator |
| 374 | Glycogen synthase kinase-3 beta |
| 375 | Alanine aminotransferase 1 |
| 376 | C-C chemokine receptor type 5 |
| 377 | Calpain-1 catalytic subunit |
| 378 | Inactive caspase-12 |
| 379 | E3 ubiquitin-protein ligase TRIM63 |
| 380 | F-box only protein 32 |
| 381 | Glucose-6-phosphate translocase |
| 382 | Phospholipase A2, membrane associated |
| 383 | Histone acetyltransferase KAT5 |
| 384 | C-C motif chemokine 20 |
| 385 | Thioredoxin |
| 386 | Neprilysin |
| 387 | Serum paraoxonase/arylesterase 1 |
| 388 | Extracellular superoxide dismutase [Cu-Zn] |
| 389 | Protein CutA |
| 390 | Guanylate cyclase soluble subunit beta-1 |
| 391 | Metallothionein-3 |
| 392 | Collagen alpha-2(I) chain |
| 393 | Ornithine decarboxylase |
| 394 | Corticoliberin |
| 395 | Metabotropic glutamate receptor 1 |
| 396 | NAD(P)H dehydrogenase [quinone] 1 |
| 397 | Kv channel-interacting protein 2 |
| 398 | Metalloproteinase inhibitor 3 |
| 399 | Metalloproteinase inhibitor 4 |
| 400 | Stromelysin-1 |
| 401 | DNA (cytosine-5)-methyltransferase 1 |
| 402 | Glutamate decarboxylase 1 |
| 403 | Calcium/calmodulin-dependent protein kinase kinase 2 |
| 404 | Protein fosB |
| 405 | cAMP-specific 3',5'-cyclic phosphodiesterase 4D |
| 406 | Pantetheinase |
| 407 | Collagenase 3 |
| 408 | P2Y purinoceptor 12 |
| 409 | Tissue factor |
| 410 | Calcium-activated potassium channel subunit alpha-1 |
| 411 | Dihydrofolate reductase |
| 412 | Adiponectin |
| 413 | Neuronal acetylcholine receptor subunit alpha-6 |
| 414 | Activity-regulated cytoskeleton-associated protein |
| 415 | Opioid-binding protein/cell adhesion molecule |
| 416 | Beta-1 adrenergic receptor |
| 417 | Leptin |
| 418 | Poliovirus receptor-related protein 1 |
| 419 | Integrin alpha-M |
| 420 | Integrin alpha-L |
| 421 | Neuronal acetylcholine receptor subunit alpha-7 |
| 422 | Nuclear receptor subfamily 4 group A member 1 |
| 423 | Retinoic acid receptor RXR-beta |
| 424 | Proenkephalin-A |
| 425 | High affinity nerve growth factor receptor |
| 426 | Integrin beta-1 |
| 427 | Orexin |
| 428 | Acyl-CoA-binding protein |
| 429 | Integrin alpha-2 |
| 430 | Delta-type opioid receptor |
| 431 | Neuropeptide Y |
| 432 | Baculoviral IAP repeat-containing protein 2 |
| 433 | Sialin |
| 434 | Interleukin-5 |
| 435 | Interleukin-13 |
| 436 | Transforming growth factor beta-3 |
| 437 | Prolactin |
| 438 | Transient receptor potential cation channel subfamily V member 1 |
| 439 | Transient receptor potential cation channel subfamily V member 4 |
| 440 | Nuclear factor erythroid 2-related factor 2 |
| 441 | Thioredoxin reductase 1, cytoplasmic |
| 442 | Interferon regulatory factor 3 |
| 443 | Toll-like receptor 4 |
| 444 | Interferon beta |
| 445 | Trans-cinnamate 4-monooxygenase |
| 446 | Protein kinase C alpha type |
| 447 | Protein kinase C gamma type |
| 448 | Protein kinase C zeta type |
| 449 | Serine/threonine-protein kinase mTOR |
| 450 | Phosphatidylcholine-sterol acyltransferase |
| 451 | Farnesyl pyrophosphate synthase |
| 452 | Pituitary adenylate cyclase-activating polypeptide |
| 453 | Proteasome assembly chaperone 1 |
| 454 | Dual specificity mitogen-activated protein kinase kinase 4 |
| 455 | Caspase-1 |
| 456 | Cytochrome P450 2C9 |
| 457 | 5-hydroxytryptamine receptor 3A |
| 458 | 5'-AMP-activated protein kinase subunit gamma-2 |
| 459 | 26S proteasome non-ATPase regulatory subunit 3 |
| 460 | Mothers against decapentaplegic homolog 2 |
| 461 | Thrombospondin-1 |
| 462 | Cadherin-1 |
| 463 | Microtubule-associated protein 1B |
| 464 | Muscarinic acetylcholine receptor M1 |
| 465 | Tubulin alpha-1A chain |
| 466 | Tumor necrosis factor receptor superfamily member 1A |
| 467 | 6-phosphofructo-2-kinase/fructose-2,6-biphosphatase 4 |
| 468 | Pyruvate kinase isozymes R/L |
| 469 | Peroxisome proliferator-activated receptor delta |
| 470 | Plasminogen |
| 471 | Cell-death-related nuclease 7 |
| 472 | Trypsin-1 |
| 473 | Cholecystokinin |
| 474 | Cbp/p300-interacting transactivator 1 |
| 475 | Fatty acid-binding protein, liver |
| 476 | Retinol-binding protein 2 |
| 477 | BDNF/NT-3 growth factors receptor |
| 478 | C-reactive protein |
| 479 | Pancreas/duodenum homeobox protein 1 |
| 480 | Solute carrier family 2, facilitated glucose transporter member 2 |
| 481 | Anthrax toxin receptor 2 |
| 482 | Peptidyl-glycine alpha-amidating monooxygenase |
| 483 | Acyl-CoA desaturase |
| 484 | Mitochondrial uncoupling protein 3 |
| 485 | Cholesteryl ester transfer protein |
| 486 | Peptide YY |
| 487 | Sterol O-acyltransferase 1 |
| 488 | Glutamyl aminopeptidase |
| 489 | Aspartyl aminopeptidase |
| 490 | Sodium-dependent noradrenaline transporter |
| 491 | Integrin alpha-6 |
| 492 | Lysosomal alpha-glucosidase |
| 493 | NADPH oxidase 4 |
| 494 | Runt-related transcription factor 2 |
| 495 | DNA-binding protein inhibitor ID-1 |
| 496 | Bone morphogenetic protein 4 |
| 497 | Alpha-actinin-1 |
| 498 | Troponin T, cardiac muscle |
| 499 | Steroid hormone receptor ERR1 |
| 500 | Nuclear respiratory factor 1 |
| 501 | Protransforming growth factor alpha |
| 502 | Cytochrome b reductase 1 |
| 503 | Solute carrier family 23 member 2 |
| 504 | Oxidized low-density lipoprotein receptor 1 |
| 505 | Resistin |
| 506 | 6-phosphogluconate dehydrogenase, decarboxylating |
| 507 | N-acylglucosamine 2-epimerase |
| 508 | Transcription factor A, mitochondrial |
| 509 | B-lymphocyte antigen CD20 |
| 510 | Tenascin |
| 511 | Collagen alpha-2(IV) chain |
| 512 | N-glycosylase/DNA lyase |
| 513 | Retinal guanylyl cyclase 2 |
